# Association of *TCF7L2*, *CASC8* and *GREM1* Polymorphisms in Patients with Colorectal Cancer and Type II Diabetes Mellitus

**DOI:** 10.3390/genes13081297

**Published:** 2022-07-22

**Authors:** Anca Florentina Mitroi, Nicoleta Leopa, Eugen Dumitru, Costel Brînzan, Cristina Tocia, Andrei Dumitru, Răzvan Cătălin Popescu

**Affiliations:** 1 Department of Pathology, Emergency Hospital of Constanța, 900591 Constanta, Romania; ank_mitroi@yahoo.com (A.F.M.); branzancostel@yahoo.com (C.B.); 2 CEDMOG Center, Ovidius University of Constanta, 900591 Constanta, Romania; eugen.dumitru@yahoo.com; 3 Faculty of Medicine, Ovidius University of Constanta, 900470 Constanta, Romania; cristina.tocia@yahoo.com (C.T.); dr.andreidumitru@gmail.com (A.D.); razvanpop2000@yahoo.com (R.C.P.); 4 Department of General Surgery, Emergency Hospital of Constanța, 900591 Constanta, Romania; 5 Department of Gastroenterology, Emergency Hospital of Constanța, 900591 Constanta, Romania

**Keywords:** colorectal, cancer, diabetes, *TCF7L2*, *CASC8*, *GREM1*, rs7903146, rs6983267, rs16969681

## Abstract

Background: The aim of the study is to explore the association between the *TCF7L2* rs7903146, *CASC8* rs6983267 and *GREM1* rs16969681 polymorphisms in patients diagnosed with type 2 diabetes mellitus (T2DM) and colorectal cancer. Methods: Sixty individuals were enrolled in this case-control study: thirty with colorectal cancer and type II diabetes mellitus (T2DM) and thirty healthy control individuals. Real-time PCR was used to determine the genotypes of *TCF7L2* rs7903146, *CASC8* rs 6983267 and *GREM1* rs16969681 in patients with CRC and T2DM and in patients without T2DM and CRC. The Hardy–Weinberg equilibrium was determined in the control group for the genotype distribution of every polymorphism. Results: People carrying the TT genotype of rs7903146, rs6983267 and rs1696981 had a significant association with T2DM and CRC. Moreover, the people with the TT genotype of rs1696981 had a greater risk for T2DM and CRC (OR = 7, CI 0.397–23.347). Conclusions: *TCF7L2* rs7903146, *CASC8* rs6983267 and *GREM1* rs16969681 could be risk factors for the association of T2DM with CRC.

## 1. Introduction

Colorectal cancer (CRC) is a malignancy with several possible risk factors. It is a heterogeneous disease that can be associated with abnormalities in various molecular pathways, with numerous studies showing that environmental factors and genetic susceptibility place certain individuals at a higher risk of developing CRC [1,2,3].

Epidemiological studies suggest a link between type II diabetes mellitus (T2DM) and CRC; diabetes promotes CRC carcinogenesis through complex processes and is considered an independent risk factor for cancer in general and for CRC in particular, contributing to a higher mortality rate for these diseases [4,5]. T2DM has been associated with increased CRC risk (20–40%) [6]. CRC and DM have in common some general risk factors such as obesity, which is prevalent in sedentary populations where a Western lifestyle is prevalent [7,8]. The risk factors of DM overlap with those of CRC and, therefore, it can be assumed that the genetic variants underlying DM could also influence susceptibility to CRC.

Other evidence is derived from preclinical study and genome-wide association study (GWAS) data that demonstrate that CRC and complications of T2DM may have common pathogenic pathways, as well as an abnormal microbiota, inflammatory mediators and transformed iron metabolisms, some of them reducing to Wnt/β-catenin signaling and mIR-21 [1,2,9]. GWAS have identified susceptibility genes for CRC and DM, such as *TCF7L2, CASC8* and *GREM1* [1,9].

The transcription factor 7-like 2 (*TCF7L2*) gene, 10q25.2–q25.3, is a transcription factor and β-catenin transcription partner in the Wnt signaling pathway that represses gene transcription in the absence of β-catenin after DNA-binding and also promotes miR-21 expression [9,10,11]. There are studies that have confirmed an association between *TCF7L2* rs7903146 and T2DM [12,13,14,15], and a weak association with CRC [16,17,18]. Another polymorphism, rs6983267, is located in Cancer Susceptibility Candidate 8 Noncoding (CASC8) 8q24.21 and the risk allele promote stronger *TCF7L2* binding, facilitating Wnt signaling [9,19]. A *GREM 1* SNP, 15q13.3, rs1696968, is also associated with CRC susceptibility and facilitates *TCF7L2* binding to DNA, leading to an increase in gene expression [20,21].

The aim of this study is to investigate the association of rs7903146, rs6983267, and rs16969681 polymorphisms with the risk of CRC in patients with T2DM.

## 2. Materials and Methods

### 2.1. Study Group

A case–control study was conducted among adult South-Eastern Romanian patients (*n* = 30) diagnosed with T2DM and with positive colonoscopic results for malignancy, histologically confirmed as CRC and prospectively admitted for elective surgery to the Surgery Department of Constanta County Clinical Emergency Hospital between September 2020 and September 2021. The control subjects (*n* = 30) included people in good health condition (no medical history and with negative colonoscopic preventive examination results) that were recruited in the same period and were frequency-matched to cases based on number, age and sex. Patients with familial adenomatous polyposis, hereditary nonpolyposis CRC, inflammatory bowel disease, or any cancer personal history were excluded from the study. According to the WHO criteria, T2DM is characterised by an inability of the body to produce insulin properly and by a plasma glucose concentration of ≥7.0 mmol/L [22].

CRC lesions were treated using laparoscopic or open surgery. The pathological stage, size and localization of the tumor were recorded. Demographic and clinical data of the participants were recorded, and included age, gender, alcohol consumption and smoking status (according to the National Institute on Alcohol Abuse and Alcoholism [23] and the Center for Disease Control and Prevention [24]), and body mass index (BMI) was calculated. Blood samples (glucose, haemoglobin A1c, uric acid, creatinine, CEA, CA 19-9) were collected after overnight fasting.

### 2.2. Genomic DNA Purification

Peripheral blood collected in specific vacutainers (with ethylenediaminetetraacetic acid) was used for genomic DNA extraction with the GeneJET Genomic DNA Purification Kit (ThermoScientific Baltics, Vilnius, Lithuania), according to the manufacturer protocol.

The purity and yield of the DNA samples were quantified using ultraviolet absorbance at 260/280 nm using a NanoDrop One™ Spectrophotometer (Thermo Fisher Scientific, Madison, WI, USA), where a ratio of A260/A280 = 1.7–2.0 and A260/A230 > 2 was considered acceptable. The concentration of the DNA samples was measured using a Qubit^®^ 3.0 Fluorometer (Thermo Fisher Scientific, Kuala Lumpur, Malayasia) and a Qubit RNA HR (high-range) Assay Kit.

### 2.3. Genotyping

SNPs of *TCF7L2* (rs7903146, C/T), *CASC8* (rs6983267, G/T), and *GREM1* (rs1696981, C/T) were identified using a real-time PCR method based on the ready-made TaqMan^®^ Genotyping Master Mix (Applied Biosystems, Waltham, MA, USA) and 20× SNP Genotyping Assay (Applied Biosystems) containing target-specific oligonucleotides labeled with a reporter dye at the 5′ end of each probe; VIC dye was linked to the 5′ end of the Allele 1 probe and FAM dye was linked to the 5′ end of the Allele 2 probe (Table 1). The DNA concentration was set between 1 and 10 ng per 10 μL of RT-PCR reaction. Briefly, each 10 μL of RT-PCR reaction consisted of 5 μL TaqMan Genotyping Master Mix (2×), 0.5 μL of TaqMan Genotyping Assay mix (20×), and 4.5 μL of DNA. Samples were incubated in a 7500 Fast Real-Time System (Applied Biosystems) with the following cycle conditions: 95 °C for 10 min, 95 °C for 15 s, and 60 °C for 1 min. The last two steps of denaturing and annealing/extension were repeated 40 times. Allelic discrimination was made with the help of 7500 Fast Real-Time PCR software, version 2.3.

### 2.4. Statistical Analysis

For statistical analysis, SPSS version 28.0 was used (IBM, Armonk, NY, USA). The results are presented as a median with a range or mean ± standard deviation, with categorical variables expressed as counts. The Hardy–Weinberg equilibrium was determined using GeneCalc software at the level of significance of 0.05. A χ^2^ test was used to establish the link between genetic variants and disease status. Odds ratios and 95% CIs were estimated. Multivariate logistic regression analysis was used for association analyses with adjustments for BMI and age. Comparisons of clinical parameters of different genotypes among patients with T2DM, CRC and healthy controls were assessed by one-way analysis of variance and the least significant difference test. *p* < 0.05 was considered to indicate a statistically significant difference.

## 3. Results

### 3.1. General Characteristics of Patients

Patients with T2DM and CRC were age-matched (within 5 years) with the control participants. Table 2 summarizes selected characteristics of the patients and controls. The mean age of the CRC and T2DM patients was 69.90 ± 8.36 years, with a mean body mass index (BMI) of 30.75 ± 3.90 kg/m^2^. The average age of the control subjects was 63.90 ± 11.9 years, with a mean BMI of 26.31 ± 5.23 kg/m^2^. There were no significant differences in mean age, sex, or the numbers of moderate alcohol drinkers between the two groups. There was a significant difference with respect to BMI, current or former smokers, glucose, systolic blood pressure, diastolic blood pressure, creatinine and uric acid in patients with CRC and T2DM than in the controls (*p* < 0.05). The time elapsed between the diagnosis of T2DM and that of CRC was between 1 and 14 years, with a mean of 7.07 ± 3.903.

The anatomopathological characteristics of patients with CRC and T2DM are shown in Table 3. The mean value of the tumor markers is above the normal limit (CEA > 5 ng/mL, CA19-9 > 27 U/mL). The most common site of cancer was the left colon (46.7%) and the rectum (30%). Most of the patients in the study were diagnosed in stage III (40%).

### 3.2. Genotype Distribution of rs7903146, rs6983267 and rs1696981 in Case and Control Groups

The genotype distribution in control groups for rs7903146, rs6983267 and rs16969681 were consistent with the Hardy–Weinberg law at the level of significance of 0.05. Univariate and multivariate analyses were performed for the genotype and allele frequencies of CRC in the T2DM patients and the control subjects and are summarized in Table 4 for the selected SNPs (rs7903146, rs6983267, and rs16969681). Compared with the TT genotype, the CC + CT genotypes demonstrated a significant association with the risk of CRC and T2DM in the univariate analysis for rs7903146 (*p* = 0.003) and rs16969681 (*p* = 0.009). This significant association was maintained after adjusting for age and BMI for rs7903146 (*p* = 0.021). Among the various parameters studied, GG + GT/TT were significantly different between the cases and controls for rs6983267 (*p* = 0.026) in the univariate analysis.

Genotype and allele distribution and the analysis of the association of rs7903146 of the *TCF7L2* gene in subjects with CRC and T2DM and in the controls are shown in Table 5. The CC, CT, and TT genotype frequencies were 26.7%, 43.3%, and 30%, respectively, in subjects with CRC and T2DM, and were 60%, 36.7%, and 3.3%, respectively, in the control subjects. The CT and TT genotypes were more frequent in subjects with CRC and T2DM than in the controls. The CC genotype was more frequent in the controls than in the subjects with CRC and T2DM. The risk of T2DM and CRC was lower in the heterozygous (CT) genotype group, with an odds ratio of 0.222 (95% CI 0.065–0.754, *p* = 0.039), than in the homozygous (TT) genotype group, which had an odds ratio of 2.042 (95% CI 0.395–10.553, *p* = 0.011). Moreover, the T allele was more frequent in the cases than in the controls and might significantly increase the occurrence risk of T2DM and CRC compared with the C allele (OR = 3.865, 95%CI = 1.743–8.567).

Genotype and allele distribution and the analysis of the association of rs6983267 of the *CASC8* gene in subjects with CRC and T2DM and in the controls are shown in Table 6. The GG, GT, and TT genotype frequencies were 30%, 40%, and 30%, respectively, in subjects with CRC and T2DM, and were 30%, 53.3%, and 16.7%, respectively, in the control subjects. The TT genotype had a lower frequency in the controls than in the subjects with T2DM and CRC. The GT genotype was more frequent in the controls than in subjects with CRC and T2DM and the GG genotype was evenly distributed in the two groups. The results indicate a low-significance association in the homozygous (TT) genotype (*p* = 0.052). There was no significant distribution of the G and T alleles between the two groups, which indicate that the presence of one T allele does not increase susceptibility for T2DM and CRC.

Genotype and allele distribution and the analysis of the association of rs16969681 of the *GREM1* gene in the subjects with CRC and T2DM and in the controls are shown in Table 7. The CC, CT, and TT genotype frequencies were 50%, 23.3%, and 28.7%, respectively, in subjects with CRC and T2DM, and were 63.3%, 23.3%, and 13.3%, respectively, in the control subjects. The TT genotype was more frequent in subjects with CRC and T2DM than in the controls. The CC genotype was more frequent in the controls than in the subjects with CRC and T2DM and the CT genotype was evenly distributed in the two groups. The results indicate a significant association in the homozygous (TT) genotype (*p* = 0.047). For rs16969689, there was also no significant distribution of the C and T alleles between the two groups, which indicates that the presence of only one risk allele is not important for susceptibility to T2DM and CRC.

**Figure 1:**
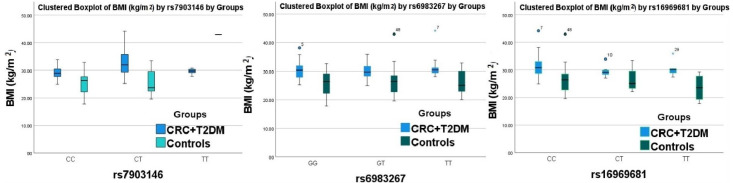
Graphical model represents the genotype distribution by BMI for patients with CRC, T2DM and controls.

Regardless of the SNP, obesity was associated with CRC and DM. When focusing on people with a BMI ≥ 30 kg/m^2^ and the TT genotype of the rs6983267 and rs16969681 genes, there was an increased frequency of cases in patients with CRC and T2DM compared to the control group (Figure 1).

## 4. Discussion

T2DM and CRC are among the most frequent causes of death worldwide [22,24]. The link between DM and cancer has been recognized by the American Diabetes Association (ADA) since the 2010 consensus guidelines and in the 2022 Standards of Medical Care in Diabetes, which state that diabetes is associated with an increased risk of cancer, and CRC is one of them [25,26]. This association may be linked with shared risk factors between T2DM and cancer (older age, obesity and physical activity), but may also be due to diabetes-related factors such as diabetes-related physiology or treatment [26].

Regarding other published studies which investigate genetic links between T2DM and CRC in different multi-ethnic populations [8,27], in our study, all individuals were of Romanian origin, and we selected the SNPs from a GWAS conducted in a Caucasian population, with the association replicated in a GWAS and meta-analysis more recently released than in mentioned studies. From the selected SNPs, only TCF7L2 rs7903146 overlapped with the outlined studies, and is a well-known factor of association between T2DM and CRC. The other two SNPs were selected because of their interaction and binding with *TCF7L2*, and are well-documented SNPs for CRC risk.

A common molecular pathway for T2DM and CRC is the Wnt/β-catenin signaling pathway. In the absence of Wnt signaling, APC attached glycogen synthase kinase 3-β (GSK-3β) and phosphorylated β-catenin to prepare it for ubiquitin–proteosome degradation. In the absence of nuclear β-catenin, a transcription factor from the *TCF* family, such as *TCF7L2*, can interact with transcriptional inhibitors and repress transcription. In CRC, *APC* is frequently mutated and, in DM, Wnt signaling is activated. Wnt signaling prevents β-catenin degradation, enabling nuclear migration where it promotes the transcription of genes implicated in cell proliferation.

*TCF7L2* is a transcription factor in the Wnt signaling pathway. It contains a DNA- binding domain, is implicated in the regulation of cell proliferation and differentiation, and maintains the stability of plasma glucose [28,29]. *TCF7L2* is related with T2DM risk and DM complications such as diabetic nephropathy, and is also a susceptibility locus for CRC [12,15,30]. In the present study, the genotype distribution of the *TCF7L2* rs7903146 polymorphism was correlated with susceptibility for CRC and DM in our population. The data show that the TT genotype frequency, and also the T allele, were significantly higher in patients with CRC and T2DM and the risk of CRC and T2DM was also significantly higher for the TT genotype. A previous study has also shown an association between T2DM and a higher risk of CRC for patients with the risk allele of *TCF7L2* rs7903146, and several of them have shown an independent association with T2DM and CRC, suggesting that the risk allele is likely to have a more important effect on colon tissue than in the pancreatic islet [8,27,28]. The Wnt pathway is frequently implicated in the etiology and pathogenesis of CRC, and *TCF7L2* undergoes changes in CRC.

Another CRC-associated polymorphism, rs6983267, located in chromosome 8q24, is situated at a *TCF7L2* binding site and the risk allele determines an intense binding of *TCF7L2*, facilitating Wnt signaling [9,19]. Our study showed a low-significance association OR 1.33 (CI 0.139–12.818, *p* = 0.05) for the risk genotype with T2DM and CRC. The other studies have shown that rs6983267 within the 8q24 region is one of the strongest genetic risk factors for the development of CRC [31]. Due to interactions with *TCF7L2*, we wanted to investigate the association between CRC and T2DM for this variant. CASC8, rs6983267, is also associated with increased susceptibility to prostate cancer, in addition to CRC, but the molecular mechanism of association is under investigation [31].

The *GREM1* rs16969681 also demonstrated a significant association with the TT genotype and T2DM and CRC, with an OR of 7 (CI 0.397–23.347, *p* = 0.047). GWASs demonstrate that *GREM 1* rs1696968 is an important risk factor for CRC or advanced adenomas in the European population [9]. Other studies propose *GREM1* as an important mediator for diabetic kidney disease, and due to its interaction with *TCF7L2*, which facilitates its DNA binding, it can be considered one of the common genetic factors for T2DM and CRC [9,32]. The *GREM1*, rs16969681, stimulates *TCF7L2* binding to DNA, determining an increase in *GREM1* gene expression. The gene product, Gremlin, activates kidney damage in T2DM and cell migration in CRC [9].

Interestingly, our study showed an increased frequency of association between high BMI and risk genotypes for rs6983267 and rs16969681 in patients with CRC and T2DM. Taken together, our results with the results of other cohort studies suggest that BMI could be an independent risk factor for the association between T2DM and the risk of CRC [8,27,33].

## 5. Conclusions

In summary, *TCF7L2* rs7903146, *CASC8* rs6983267 and *GREM1* rs1696968 were significantly correlated in our study with T2DM and CRC. There is no other study, to our knowledge, that investigates the association between T2DM and CRC for rs6983267 and rs1696968. In the present study, there are several limitations such as sample size and interaction between genetic and environmental factors that were ignored. Therefore, further research should be conducted to verify this conclusion.

## Figures and Tables

**Table 1:** VIC/FAM Sequences of SNP Genotyping Assay.

SNP ID	VIC/FAM Sequences
rs7903146	TAGAGAGCTAAGCACTTTTTAGATA[C/T]TATATAATTTAATTGCCGTATGAGG
rs6983267	GTCCTTTGAGCTCAGCAGATGAAAG[G/T]CACTGAGAAAAGTACAAAGAATTTT
rs1696981	TTTCTTTTTATCTTGATATCTTGCA[C/T]GCGGCCTAACAAAGGCAATAATAAC

**Table 2:** Clinical and biochemical characteristics of subjects with CRC + T2DM and controls.

Variable	CRC + T2DM (*n* = 30)	Controls (*n* = 30)	*p*-Value
Age (years)	69.90 ± 8.36	63.90 ± 11.9	0.250
Sex *			0.193
Male	19 (63.3)	14 (46.7)	
Female	11 (36.7)	16 (53.3)	
BMI (kg/m^2^)	30.75 ± 3.90	26.31 ± 5.23	0.036
Current of former smokers *	9 (30)	7 (23.3)	0.049
Moderate alcohol consumption *	6 (20)	7 (23.3)	0.072
Glu (mg/dL)	143.29 ± 14.59	83.33 ± 12.87	<0.001
Blood HbA1c (%)	6.87 ± 0.96	5.16 ± 0.33	<0.001
SBP (mmHg)	136.98 ± 16.84	122.48 ± 11.26	0.042
DBP (mmHg)	81.68 ± 0.97	69.88 ± 0.77	<0.001
UA (mg/dL)	39.7 ± 16.5	16.9 ± 9.3	0.018
Cr (mg/dL)	1.13 ± 0.11	0.58 ± 0.26	0.029

Variables are expressed as mean ± SD (standard deviation), unless indicated otherwise. * Number of cases with percentages in parentheses. CRC—colorectal cancer; T2DM—type 2 diabetes mellitus; BMI—body mass index; smoker—smoking of ≥10 cigarettes daily; alcohol consumption ≥ 1 drink per day for women and ≥2 drinks per day for men; Glu—glucose; HbA1c—haemoglobin A1c; SBP—systolic blood pressure; DBP—diastolic blood pressure; UA—uric acid; Cr—creatinine.

**Table 3:** Tumor characteristics of patients with CRC and T2DM.

Variable	Patients with CRC and T2DM (*n* = 30)	Percentage (%)
CEA (ng/mL) *	50.51	
CA19-9 (U/mL) *	43.15	
Tumor site		
Right colon	7	23.3
Left colon	14	46.7
Rectum	9	30
Disease stage TNM		
Stage I	8	26.7
Stage II	8	26.7
Stage III	12	40
Stage IV	2	6.7

CRC—colorectal cancer; T2DM—type 2 diabetes mellitus; CEA—cancer embryonic antigen; CA19-9—carbohydrate antigen 19-9; TNM—tumor node metastasis. * values are median.

**Table 4:** Univariate and multivariate analyses for CRC and T2DM patients and control subjects.

SNP		Univariate Analysis			Multivariate Analysis	
		CRC + T2DM *n* = 30	Controls *n* = 30	*p* Value	OR [95%CI]	*p* Value
rs7903146	CC + CT TT	21 (70) 9 (30)	29 (96.7) 1 (3.3)	0.003	0.080 [0.009–0.685]	0.021
rs6983267	GG + GT TT	21 (70) 9 (30)	25 (83.3) 5 (16.7)	0.026	2.143 [0.622–7.387]	0.227
rs16969681	CC + CT TT	22 (73.3) 8 (26.7)	26 (86.7) 4 (13.3)	0.009	2.364 [0.627–8.917]	0.204

Variables are expressed as number of cases with percentages in parentheses. SNP—single-nucleotide polymorphism; CRC—colorectal cancer; T2DM—type 2 diabetes mellitus; OR—odds ratio; CI—confidence interval.

**Table 5:** Genotype and allele distribution and analysis of the association of rs7903146 of *TCF7L2* in subjects with CRC + T2DM and controls.

Genotype	CRC + T2DM (*n* = 30)	Controls (*n* = 30)	OR	95% CI	*p*-Value
CC (%)	8 (26.7%)	18 (60%)	Reference		
CT (%)	13 (43.3%)	11 (36.7%)	0.222	0.065–0.754	0.039
TT (%)	9 (30%)	1 (3.3%)	2.042	0.395–10.553	0.011
C (%)	29 (48.3%)	47 (78.3%)		Reference	
T (%)	31 (51.7%)	13 (21.7%)	3.865	1.743–8.567	0.001

CRC—colorectal cancer; T2DM—type 2 diabetes mellitus; OR—odds ratio; 95%CI—95% confidence interval.

**Table 6:** Genotype and allele distribution and analysis of the association of rs6983267 of *CASC8* in subjects with CRC + T2DM and controls.

Genotype	CRC + T2DM (*n* = 30)	Controls (*n* = 30)	OR	95% CI	*p*-Value
GG (%)	9 (30%)	9 (30%)	Reference		
GT (%)	12 (40%)	16 (53.3%)	3.000	0.586–15.362	0.586
TT (%)	9 (30%)	5 (16.7%)	1.333	0.139–12.818	0.052
G (%)	30 (50%)	34 (56.7%)		Reference	
T (%)	30 (50%)	26 (43.3%)	0.765	0.373–1.569	0.464

CRC—colorectal cancer; T2DM—type 2 diabetes mellitus; OR—odds ratio; 95%CI—95% confidence interval.

**Table 7:** Genotype and allele distribution and analysis of the association of rs16969681 of *GREM1* in subjects with CRC + T2DM and controls.

Genotype	CRC + T2DM (*n* = 30)	Controls (*n* = 30)	OR	95% CI	*p*-Value
CC (%)	15 (50%)	19 (63.3%)	Reference		
CT (%)	7 (23.3%)	7 (23.3%)	6.250	0.615–63.538	0.109
TT (%)	8 (28.7%)	4 (13.3%)	7.000	0.397–23.347	0.047
C (%)	37 (61.7%)	45 (75%)		Reference	
T (%)	23 (38.3%)	15 (30%)	0.536	0.245–1.173	0.116

CRC—colorectal cancer; T2DM—type 2 diabetes mellitus; OR—odds ratio; 95%CI—95% confidence interval.

## Data Availability

The data presented in this study are available on request from the authors.

## References

[1] Peters U., Jiao S., Schumacher F.R., Hutter C.M., Aragaki A.K., Baron J.A., Berndt S.I., Bézieau S., Brenner H., Butterbach K. (2013). Colon Cancer Family Registry and the Genetics and Epidemiology of Colorectal Cancer Consortium. Identification of Genetic Susceptibility Loci for Colorectal Tumors in a Genome-Wide Meta-analysis. Gastroenterology.

[2] Lu Y., Kweon S.-S., Tanikawa C., Jia W.-H., Xiang Y.-B., Cai Q., Zeng C., Schmit S.L., Shin A., Matsuo K. (2019). Large-Scale Genome-Wide Association Study of East Asians Identifies Loci Associated with Risk for Colorectal Cancer. Gastroenterology.

[3] Popescu R.C., Tocia C., Brînzan C., Cozaru G.C., Deacu M., Dumitru A., Leopa N., Mitroi A.F., Nicolau A., Dumitru E. (2021). Molecular profiling of the colon cancer in South-Eastern Romania: Results from the MERCUR study. Medicine.

[4] Singh S., Earle C.C., Bae S.J., Fischer H.D., Yun L., Austin P.C., Rochon P.A., Anderson G.M., Lipscombe L. (2016). Incidence of Diabetes in Colorectal Cancer Survivors. J. Natl. Cancer Inst..

[5] Agache A., Bîrligea A., Botea S., Cirstea M., Mihalache O., Mustăţea P. (2021). Assessment of the Risk of Colorectal Cancer in Patients with Diabetes Mellitus. Chirurgia.

[6] Tsilidis K.K., Kasimis J.C., Lopez D.S., Ntzani E.E., Ioannidis J.P. (2015). Type 2 diabetes and cancer: Umbrella review of meta-analyses of observational studies. BMJ.

[7] Peeters P.J., Bazelier M.T., Leufkens H.G., de Vries F., De Bruin M.L. (2015). The risk of colorectal cancer in patients with type 2 diabetes: Associations with treatment stage and obesity. Diabetes Care.

[8] Cheng I., Caberto C.P., Lum-Jones A., Seifried A., Wilkens L.R., Schumacher F.R., Monroe K.R., Lim U., Tiirikainen M., Kolonel L.N. (2011). Type 2 diabetes risk variants and colorectal cancer risk: The Multiethnic Cohort and PAGE studies. Gut.

[9] González N., Prieto I., del Puerto-Nevado L., Portal-Nuñez S., Ardura J.A., Corton M., Fernández-Fernández B., Aguilera O., Gomez-Guerrero C., Mas S. (2017). 2017 update on the relationship between diabetes and colorectal cancer: Epidemiology, potential molecular mechanism and therapeutics implications. Oncotarget.

[10] Clevers H., Nusse R. (2012). Wnt/β-catenin signaling and disease. Cell.

[11] Lan F., Yue X., Han L., Shi Z., Yang Y., Pu P., Yao Z., Kang C. (2012). Genome-wide identification of TCF7L2/TCF4 target miRNAs revels a role for miR-21 in Wnt-driven epithelial cancer. Int. J. Oncol..

[12] Peng S., Zhu Y., Lü B., Xu F., Li X., Lai M. (2013). TCF7L2 gene polymorphisms and type 2 diabetes risk: A comprehensive and updated meta-analysis involving 121,174 subjects. Mutagenesis.

[13] Akhundova L.A., RRustamova Z., RAlibayova G., Sh Mustafayev N., MHuseynova I. (2022). Possible Role of rs7903146 Polymorphism of the Transcription Factor 7-Like 2 Gene in Genetic Predisposition to Type 2 Diabetes. Pak. J. Biol. Sci..

[14] Hameed T., Khan Z., Imran M., Ali S., Albegali A.A., Ullah M.I., Ejaz H. (2021). Associations of transcription factor 7-Like 2 (TCF7L2) gene polymorphism in patients of type 2 diabetes mellitus from Khyber Pakhtunkhwa population of Pakistan. Afr. Health Sci..

[15] Bride L., Naslavsky M., Lopes Yamamoto G., Scliar M., Pimassoni L.H., Sossai Aguiar P., de Paula F., Wang J., Duarte Y., Passos-Bueno M.R. (2021). TCF7L2 rs7903146 polymorphism association with diabetes and obesity in an elderly cohort from Brazil. PeerJ.

[16] Zhang M., Tang M., Fang Y., Cui H., Chen S., Li J., Xiong H., Lu J., Gu D., Zhang B. (2018). Cumulative evidence for relationships between multiple variants in the VTI1A and TCF7L2 genes and cancer incidence. Int. J. Cancer.

[17] Rosales-Reynoso M.A., Arredondo-Valdez A.R., Juárez-Vázquez C.I., Wence-Chavez L.I., Barros-Núñez P., Gallegos-Arreola M.P., Flores-Martínez S.E., Morán-Moguel M.C., Sánchez-Corona J. (2016). TCF7L2 and CCND1 polymorphisms and its association with colorectal cancer in Mexican patients. Cell Mol. Biol..

[18] Karimi F., Amiri-Moghaddam S.M., Bagheri Z., Bahrami A.R., Goshayeshi L., Allahyari A., Mirsadraee M., Fanipakdel A., Bari A., Emadi-Torghabeh A. (2021). Investigating the association between rs6983267 polymorphism and susceptibility to gastrointestinal cancers in Iranian population. Mol. Biol. Rep..

[19] Tuupanen S., Turunen M., Lehtonen R., Hallikas O., Vanharanta S., Kivioja T., Björklund M., Wei G., Yan J., Niittymäki I. (2009). The common colorectal cancer predisposition SNP rs6982367 at chromosome 8q24 confers potential to enhanced WNT signaling. Nat. Genet..

[20] Lewis A., Freeman-Mills L., de la Calle-Mustienes E., Giráldez-Pérez R.M., Davis H., Jaeger E., Becker M., Hubner N.C., Nguyen L.N., Zeron-Medina J. (2014). A polymorphic enhancer near GREM1 enfluences bowel cancer risk through differemtial CDX2 and TCF7L2 binding. Cell Rep..

[21] Davis H., Irshad S., Bansal M., Rafferty H., Boitsova T., Bardella C., Jaeger E., Lewis A., Freeman-Mills L., Giner F.C. (2015). Aberrant epithelial GREM1 expression initiates colonic tumorigenesis from cells outside the stem cell miche. Nat. Med..

[22] World Health Organization, International Diabetes Federation (2006). Definition and Diagnosis of Diabetes Mellitus and Intermediate Hyperglycaemia: Report of a WHO/IDF Consultation. https://apps.who.int/iris/handle/10665/43588.

[23] National Institute on Alcohol Abuse and Alcoholism Drinking Levels Defined. https://www.niaaa.nih.gov/alcohol-health/overview-alcohol-consumption/moderate-binge-drinking.

[24] Centers for Disease Control and Prevention National Center for Health Statistics Tobacco Glossary. https://www.cdc.gov/nchs/nhis/tobacco/tobacco_glossary.htm.

[25] American Diabetes Association (2016). 3. Foundation of Care and Comprehensive Medical Evaluation. Diabetes Care.

[26] American Diabetes Association (2022). Standards of Medical Care in Diabetes-2022. Diabetes Care.

[27] Sainz J., Rudolph A., Hoffmeister M., Frank B., Brenner H., Chang-Claude J., Hemminki K., Försti A. (2012). Effect of type 2 diabetes predisposing genetic variants on colorectal cancer risk. J. Clin. Endocrinol. Metab..

[28] Grant S.F., Thorleifsson G., Reynisdottir I., Benediktsson R., Manolescu A., Sainz J., Helgason A., Stefansson H., Emilsson V., Helgadottir A. (2006). Variant of transcription factor 7-like 2(TCF7L2) gene confers risk of type 2 diaabets. Nat. Genet..

[29] Ng M.C.Y., Shriner D., Chen B.H., Li J., Chen W.M., Guo X., Liu J., Bielinski S.J., Yanek L.R., Nalls M.A. (2014). Meta-anlysis of genome wide association studies in African Americans provides inshints into the genetic architecture of type 2 diabetes. PLoS Genet..

[30] Franceschini N., Shara N.M., Wang H., Voruganti V.S., Laston S., Haack K., Lee E.T., Best L.G., MacCluer J.W., Cochran B.J. (2012). The association of genetic variants of type 2 diabetes with kidney function. Kidney Int..

[31] Pomerantz M., Ahmadiyeh N., Jia L., Herman P., Verzi M.P., Doddapaneni H., Beckwith C.A., Chan J.A., Hills A., Davis M. (2009). The 8q24 cancer risk variant rs6983267 demonstrates long interaction with MYC in colorectal cancer. Nat. Genet..

[32] McKnight A.J., Patterson C.C., Pettigrew K.A., Savage D.A., Kilner J., Murphy M., Sadlier D., Maxwell A.P., Warren 3/UK Genetics of Kidneys in Diabetes (GoKinD) Study Group (2010). A GREM1 gene variant associates with dibetic nephopaty. J. Am. Soc. Nephrol..

[33] Folsom A.R., Pankow J.S., Peacokk J.M., Bielinski S.J., Heiss G., Boerwinkle E. (2008). Variation in TCF7L2 and increase risk of colon cancer: The Atherosclerosis Risk in Communities (ARIC) Study. Diabetes Care.

